# Visual Performance Comparison Between Enhanced Monofocal and Extended Depth of Focus Intraocular Lenses: A Systematic Review and Meta-Analysis

**DOI:** 10.7759/cureus.103841

**Published:** 2026-02-18

**Authors:** Danai Barpaki, Diamantis Almaliotis, Sofia Androudi, Konstantinos T Tsaousis, Ioannis Tsinopoulos

**Affiliations:** 1 Laboratory of Experimental Ophthalmology, Aristotle University of Thessaloniki, Thessaloniki, GRC; 2 Second Department of Ophthalmology, School of Medicine, Aristotle University of Thessaloniki, Thessaloniki, GRC; 3 Department of Ophthalmology, University of Thessaly, Larissa, GRC; 4 Department of Ophthalmology, Achillopouleio General Hospital of Volos, Volos, GRC

**Keywords:** cataract, edof, enhanced monofocal, iol, vision

## Abstract

The objective of this systematic review is to directly compare enhanced monofocal and extended depth of focus (EDOF) intraocular lenses (IOLs) with respect to visual outcomes after cataract surgery. MEDLINE, CENTRAL, clinicaltrials.gov and ICTRP were searched until April 1, 2025, to retrieve studies that compared postoperative outcomes between enhanced monofocal and EDOF IOLs. The search was based on prespecified criteria for patients, intervention, comparator, outcomes and study type. The outcomes in question were uncorrected and corrected distance, intermediate and near visual acuities, and subjective glare and halo perception. The Cochrane risk of bias tool (RoB 2) was used for risk of bias assessment for randomized studies, while the Joanna Briggs Institute (JBI) critical appraisal tool was used for risk of bias assessment for non-randomized studies. Random-effects meta-analysis was performed for uncorrected and corrected distance visual acuity.

Five studies with a total of 672 eyes were included: one randomized study, one prospective comparative case control study, one retrospective comparative study and two prospective comparative case series. All studies directly compared enhanced monofocal with EDOF IOLs. Tecnis Eyhance was the enhanced monofocal IOL in all studies, while the EDOF lenses included Tecnis Symfony in three studies, Tecnis PureSee in one study and Acrysof IQ Vivity in one study. The meta-analysis that was performed for uncorrected and corrected distance visual acuity showed that there was no significant clinical difference between the two types of IOLs (mean difference: -0.01 (-0.03, -0.00), 95% CI and -0.01 (-0.02, -0.00), 95% CI, respectively). The results of individual studies pertaining to the rest of the outcomes (uncorrected and distance corrected intermediate visual acuity, uncorrected and distance corrected near visual acuity, subjective glare and halo perception) were also presented.

Intermediate visual acuity improvement via cataract surgery is of particular interest contemporarily. A quantitative synthesis regarding this outcome was not feasible due to substantial heterogeneity (I^2^ > 50%), making meta-analysis inappropriate for this endpoint. Prevalence of non-randomized studies and different EDOF designs may have contributed to heterogeneity. Further research should be carried out.

## Introduction and background

Cataract is estimated to be the second most frequent cause of moderate to severe vision decline worldwide, particularly among adults over-60 years of age [1,2]. Over the last decades, along with the intraocular lens (IOL) technology, cataract surgery has evolved from a vision-restoring procedure into a refractive one, with the IOL selection increasingly based on postoperative visual performance and spectacle independence. In particular, patients’ investment in several daily activities - such as computer use, household tasks, dashboard viewing - has led to the rise of relevance of intermediate visual acuity, apart from the conventional aim to improve distance visual acuity. Consequently, IOL technologies that extend functional vision while preserving optical quality have gained substantial clinical interest [3].

In 2014, the European Market was introduced with an extended depth of focus (EDOF) IOL, with its approval by the FDA in 2016. Its structure involves a single elongated focal point, in an attempt to achieve intermediate and near vision improvement, while aiming to reduce dysphotopsias that could be experienced with multifocal IOLs’ near and far image overlap [4]. The main types of EDOF, depending on structure and function based on optic properties, are pure, hybrid multifocal and wavefront shaping EDOF IOLs. Although all of these designs aim to extend the depth of focus, their diverse optical principles mean that their visual performance may not be directly comparable [5-8].

The gap between standard monofocal and EDOF IOLs was attempted to be bridged with the introduction of enhanced monofocal IOLs. These IOLs employ modified, non-diffractive optical profiles, intended to provide extended depth of vision while maintaining the visual quality and dysphotopsia profile of a conventional monofocal IOL. Tecnis Eyhance ICB00 was the first enhanced monofocal IOL to be introduced in 2019. The difference from its conventional monofocal predecessor lies in its modified anterior surface, which creates a centripetal continuous power profile, thus leading to an extended range of vision. Recent studies showed the efficacy of Tecnis Eyhance both on distance and on intermediate visual acuity improvement [9].

There is an increasing clinical adoption of both EDOF and enhanced monofocal IOLs. However, direct comparative evidence between these two IOL types remains limited and fragmented. This study is a direct comparative systematic review and meta-analysis, synthesizing evidence from head-to-head comparisons between enhanced monofocal and EDOF IOLs. The aim of this meta-analysis is to clarify whether these two IOL types provide comparable functional outcomes pertaining to distance, intermediate and near visual acuity, and subjective quality of vision (glare and halo perception).

## Review

Methods

Eligibility Criteria

Τhe following criteria were prespecified for study selection: Patients over 18 years old who were diagnosed with unilateral or bilateral age-related cataract and underwent phacoemulsification were included. Patients with congenital, traumatic or pharmaceutically induced cataract were excluded. The intervention and the comparator investigated were the enhanced monofocal IOL - defined as a monofocal lens with a modified optical profile designed to extend depth of focus without diffractive optics - and the EDOF IOL, respectively. The studies that performed a direct comparison between these two types of IOLs were included.

The primary outcomes were uncorrected distance visual acuity (UDVA), corrected distance visual acuity (CDVA), uncorrected intermediate visual acuity (UIVA) and distance corrected intermediate visual acuity (DCIVA), assessed at three months minimum postoperatively. When longer follow-ups were reported, these data were also included. Intermediate visual acuity outcomes, in particular, were prioritized due to their clinical relevance in daily activities and their role as a key differentiating feature between enhanced monofocal and EDOF IOLs. The secondary outcomes were postoperative uncorrected near visual acuity (UNVA), distance corrected near visual acuity (DCNVA) and subjective quality-of-vision outcomes (glare and halo perception). Subjective quality-of-vision outcomes were extracted as reported in the original studies, regardless of the specific questionnaire or assessment tool used. Studies that did not provide data for the primary outcomes were excluded.

All studies that were published within the last five years in the English language and directly compared the effect of the two IOLs were included. Conference abstracts, theses and other non-published material were excluded, as well as narrative reviews and systematic reviews and meta-analyses, which would not provide primary data.

Search Strategy

The databases that were searched were MEDLINE and the Cochrane Library, starting from March 26, 2023, until April 1, 2025. Gray literature was covered with the search in Clinicaltrials.gov registry and the International Clinical Trials Registry Platform (ICTRP). The Population, Intervention, Comparison, and Outcome (PICO) elements of our clinical question served as keywords, with a combination of free text and Medical Subject Headings (MeSH) terms. The search was limited to the last five years to capture contemporary IOL technologies and surgical techniques relevant to current clinical practice. The search strategy was evaluated against the Peer Review of Electronic Search Strategies (PRESS) checklist [10].

The final search string for PubMed is presented: (((((((("Lenses, Intraocular"[Mesh]) OR (intraocular lens)) OR (intraocular lenses)) OR (IOL)) OR ("Lens Implantation, Intraocular"[Mesh])) AND (((EDOF) OR ("extended depth of focus")) OR ("enhanced depth of focus"))) AND (((enhanced intermediate monofocal) OR (tecnis eyhance)) OR (enhanced monofocal))) AND ((("Cataract"[Mesh]) OR ("Cataract Extraction"[Mesh])) OR (cataract surgery))) AND ((intermediate vision) OR (visual acuity)).

The same key words were applied in the Cochrane Library: “intraocular lens”(#1), IOL (#2), “intraocular lens implantation” (#3) (#4: #1 OR #2 OR #3); “extended depth of focus” (#5), EDOF (#6), “enhanced depth of focus” (#7) (#8: #5 OR #6 OR #7); “enhanced monofocal” (#9), “enhanced intermediate monofocal” (#10), “Tecnis Eyhance” (#11) (#12: #9 OR #10 OR #11); “cataract extraction” (#13), “cataract surgery” (#14) (#15: #13 OR #14); and “visual acuity” (#16), “intermediate vision” (#17) (#18: #16 OR #17).

The final search string combined these concepts as: #4 AND #8 AND #12 AND #15 AND #18.

Study Selection and Data Collection

Covidence reference management software (Veritas Health Innovation, Melbourne, Australia) was used for study screening. Two independent reviewers carried out the screening, firstly by excluding duplicates, then by examining the title and abstract, and then the full text [11]. Disagreements were first discussed so as to reach a consensus; a third reviewer was consulted for unresolved discrepancies.

The subsequent data extraction included: study details; baseline characteristics and measures of patients of each group with p-values, if available; IOL type; postoperative - at least three months post-op - monocular uncorrected and corrected distance, intermediate and near visual acuities; data related to subjective glare and halo perception.

Means and standard deviations for the quantitative outcomes assessed were extracted. Alternative reported statistics (confidence intervals, standard errors or p-values) were planned to be implemented, in case of absence of essential data.

Risk of Bias Assessment

Two independent reviewers performed risk of bias assessment for the selected studies. Efforts for disagreement resolution were made again initially through discussion and then with the contribution of a third reviewer, so as to achieve a consensus.

The Joanna Briggs Institute (JBI) critical appraisal tool was used to assess the risk of bias of non-randomized studies at the level of the primary outcomes. The respective modification of the tool was implemented according to the study type. The tool checklist consists of items with four possible responses ("yes," "no," "unclear," and "not applicable"). These items evaluate key methodological domains, including participant selection, comparability of groups, validity and reliability of outcome measurement, identification and management of confounding factors, adequacy of follow-up, and appropriateness of statistical analysis and reporting. The number of "yes" responses was recorded for descriptive purposes; however, risk-of-bias judgments were based on domain-level appraisal within each checklist [12].

The revised Cochrane risk-of-bias assessment tool for randomized trials (RoB 2) was used for a randomized study at the level of each primary outcome. The effect of assignment to intervention (intention-to-treat effect) was assessed, as patients were analyzed according to the IOL type to which they were randomized. Following phacoemulsification, each participant received the allocated IOL, and no post-randomization deviations or adherence issues were applicable. Therefore, the comparison between the two IOL groups was accurately reflected with the intention-to-treat framework. Randomization process, deviations from intended interventions, missing outcome data, measurement of the outcome, and selection of the reported result were assessed. Each domain was ranked as “high risk of bias,” “some concerns” and “low risk of bias” [13]. Outcome-specific judgments informed whether each outcome was considered eligible for quantitative synthesis.

Effect Measures

The unit of analysis used in this study was the eye. When studies reported outcomes for both eyes of the same patient, analyses were conducted at the eye level as presented in the original studies; eyes were treated as independent units, as per the primary publications. This approach does not account for inter-eye correlation and may underestimate variance, potentially inflating statistical significance, particularly for outcomes approaching borderline significance. Mean difference was used as a measure of treatment effect for visual acuity. Logarithm of the minimum angle of resolution (LogMAR) values were used, and a commonly used threshold of 0.1 logMAR (equivalent to one Early Treatment Diabetic Retinopathy Study (ETDRS) line or five letters) was set; values over it were considered of clinical significance. A narrative presentation of results regarding halo and glare perception followed.

Synthesis

Risk of bias for randomized studies was assessed at the outcome level; therefore, outcomes judged as low risk of bias or raising only minor concerns were considered eligible for quantitative synthesis. As far as the non-randomized studies were concerned, the prespecified threshold for synthesis eligibility was 50% or more domains rated "yes." Summary statistics tables were constructed for the results of each study, using means and standard deviations for continuous variables and the number of events out of the total number of each group for dichotomous outcomes.

Review Manager 5.4.1 (RevMan) was used for statistical synthesis and forest plot construction. A random-effects model using the DerSimonian-Laird estimator was prespecified and applied for all quantitative analyses to account for expected clinical and methodological diversity among studies. The inverse variance method was used. Regardless of heterogeneity, this model was retained, so as to maintain consistency with the prespecified protocol. Statistical heterogeneity was assessed using the Chi^2^ test (p-value < 0.1 indicating significant heterogeneity) and quantified using the I^2^ statistic, with I^2^ > 50% representing substantial heterogeneity. When heterogeneity exceeded this threshold, meta-analysis was deemed inappropriate, and results were summarized narratively [14].

Subgroup analyses by EDOF optical design were prespecified. Subgroup comparisons were to be performed only when at least two studies were available within each subgroup. For outcomes with at least three studies, a leave-one-out sensitivity analysis was planned to examine whether the overall pooled effect was driven by any single study. Each study was iteratively removed, and the meta-analysis was recalculated to assess the stability of the results.

Results

Study Selection

**Figure 1:**
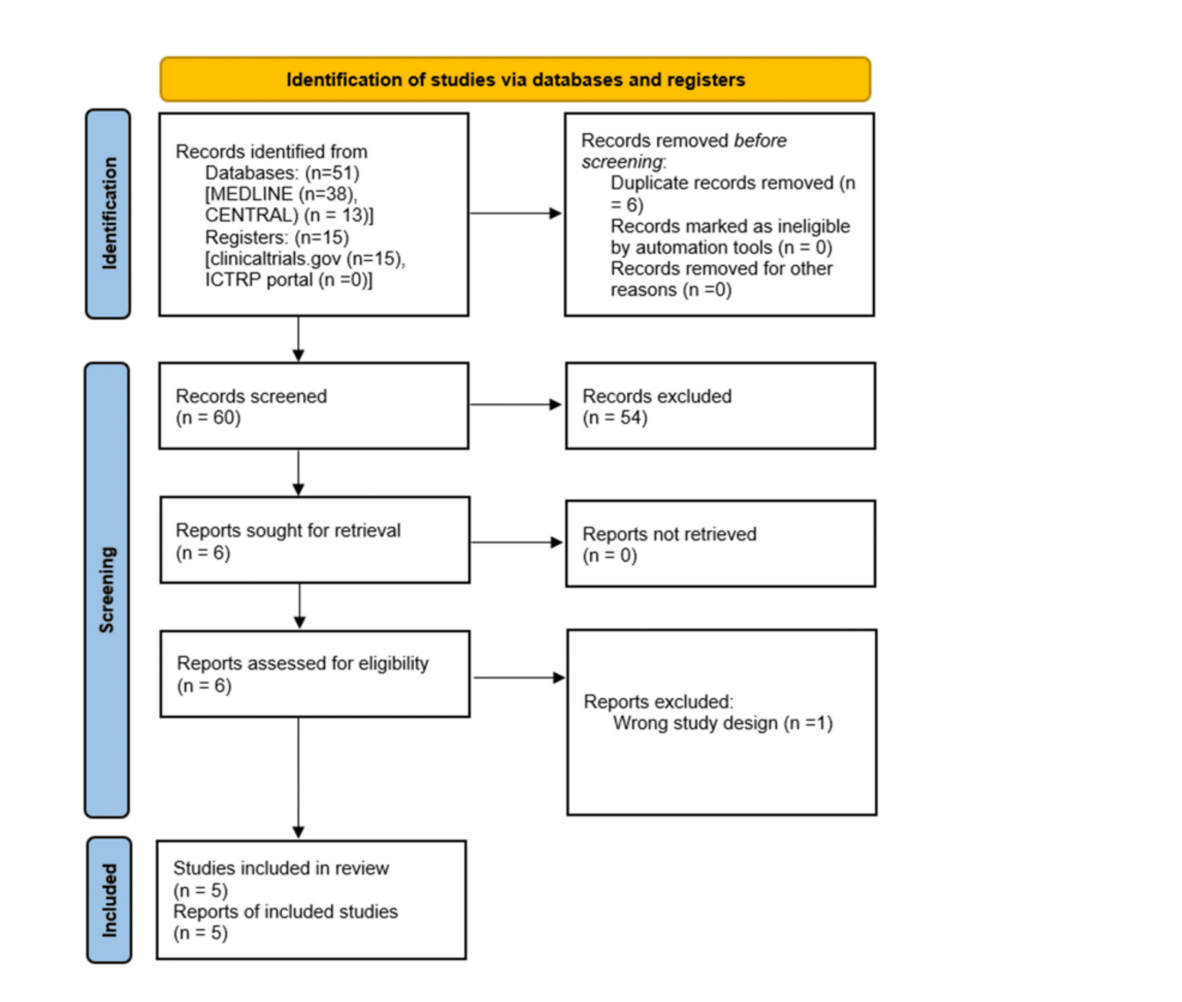
PRISMA 2020 flow diagram Visual representation of the study selection process, including duplicate removal, followed by screening of titles and abstracts and subsequent full-text assessment. PRISMA: Preferred Reporting Items for Systematic Reviews and Meta-Analyses

After applying the previously described elaborate search strategy, MEDLINE and CENTRAL databases, clinicaltrials.gov and ICTRP portal registers for clinical trials resulted in 66 studies. Six records were excluded as duplicates, and 54 were excluded by abstract and title screening. One record was excluded at full text stage due to a wrong study design (systematic review). Therefore, five studies were finally selected [15-19]. Figure 1 presents a visualization of the steps that led to the final study selection, with the Preferred Reporting Items for Systematic Reviews and Meta-Analyses (PRISMA) flow diagram [20].

Study Characteristics

Table 1 presents the main characteristics of our eligible studies. All studies directly compared enhanced monofocal and EDOF IOLs following phacoemulsification, with a minimum follow-up of three months; two studies reported six-month outcomes. Intermediate visual acuity was assessed at 66 cm, and near visual acuity at 40 cm in all studies. Visual acuity outcomes were reported in logMAR units, and complete outcome data were available.

**Table 1:** Study characteristics A summary of the characteristics of each included study was extracted, including study identification number; study design; sample size (reported as both number of eyes, representing the unit of analysis and number of patients); follow-up duration; type of enhanced monofocal and extended depth of focus (EDOF) intraocular lenses; EDOF optical design; number of eyes in each study arm; primary and secondary visual outcomes measured; and the presence or absence of halo and glare assessment [15-19]. UDVA: uncorrected distance visual acuity; CDVA: corrected distance visual acuity; UIVA: uncorrected intermediate visual acuity; UNVA: uncorrected near visual acuity

Study ID	Type of study	Sample size	Follow-up duration	Enhanced monofocal number of eyes	EDOF number of eyes	EDOF optical design	Outcomes	Glare/halo assessment
Sabur and Unsal (2023) [15]	Prospective comparative case control	76 eyes (38 patients)	3 months	Tecnis Eyhance 36	Acrysof Vivity 40	Wavefront-shaping	UDVA, CDVA, UIVA, DCIVA, UNVA, DCNVA	YES
Lee et al. (2022) [16]	Prospective comparative case series	88 eyes (44 patients)	3 months	Tecnis Eyhance 48	Tecnis Symfony ZXR00 40	Diffractive	UDVA, CDVA, UIVA, UNVA	YES
Corbelli et al. (2022) [17]	Prospective comparative case series	100 eyes (50 patients)	6 months	Tecnis Eyhance 50	Tecnis Symfony ZXR00 50	Diffractive	UDVA, CDVA, UIVA, DCIVA, UNVA, DCNVA	YES
Corbett et al. (2024) [18]	Prospective randomised study	234 eyes (117 patients)	6 months	Tecnis Eyhance 114	Tecnis PureSee 120	Refractive	CDVA, DCIVA, DCNVA	YES
Jeon et al. (2021) [19]	Retrospective comparative study	174 eyes (174 patients)	3 months	Tecnis Eyhance 102	Tecnis Symfony ZXR00 72	Diffractive	UDVA, CDVA, UIVA, UNVA	NO

Risk of Bias Assessment

**Figure 2:**
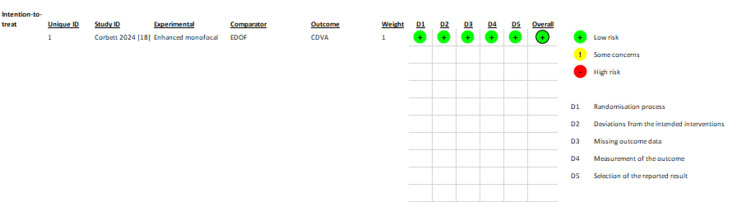
Visual summary of risk of bias assessment for the randomized study Visual representation of risk of bias assessment for the randomized study, using the RoB 2 tool [18]. Risk of bias was assessed for the primary outcome of corrected distance visual acuity (CDVA). The effect of assignment to intervention (intention-to-treat effect) was assessed, as it was appropriate for this surgical comparison. The "experimental" and "comparator" labels follow RoB 2 convention and do not imply investigational status of either IOL. Randomization process, deviations from intended interventions, missing outcome data, measurement of the outcome and selection of the reported result were assessed. All domains were rated as low risk of bias. As the same methods were used for distance corrected intermediate visual acuity (DCIVA), the risk of bias assessment was identical for both outcomes. IOL: intraocular lens; EDOF: extended depth of focus

Risk of bias assessment results are summarized in Table 2. All four non-randomized studies met the prespecified threshold for inclusion in the meta-analysis, while detailed domain-level assessments are presented in the Appendices. Figure 2 shows a visual summary of the risk of bias assessment of the randomized study, which demonstrated low risk of bias across all assessed domains for the primary outcomes.

**Table 2:** Risk of bias assessment for each study Each study is presented along with the risk of bias assessment tool that was used for its evaluation at the level of the primary outcomes, its overall appraisal and the evaluation deduction. Joanna Briggs Institute (JBI) critical appraisal tools appropriate to study design were used to assess the risk of bias of non-randomized studies. The revised Cochrane RoB 2 was used for the randomized study. Numeric JBI scores are reported descriptively; domain-level assessments informed study inclusion [15-19].

Study ID	Risk of bias assessment tool	Overall appraisal	Evaluation
Sabur and Unsal (2023) [15]	JBI Critical Appraisal Checklist for Case Control Studies	5/10	Included
Lee et al. (2022) [16]	JBI Critical Appraisal Checklist for Case Series	8/10	Included
Corbelli et al. (2022) [17]	JBI Critical Appraisal Checklist for Case Series	6/10	Included
Corbett et al. (2024) [18]	Revised Cochrane Risk-of-Bias Assessment Tool for Randomized Trials (RoB 2)	Low risk of bias	Included
Jeon et al. (2021) [19]	JBI Critical Appraisal Checklist for Cohort Studies	6/11	Included

Results of Individual Studies

Summary statistics, for each study and of all the outcomes, are presented in the tables below. Uncorrected (UDVA) and corrected (CDVA) distance visual acuity showed no statistically or clinically meaningful difference between enhanced monofocal and EDOF IOLs in any study. All mean differences were below the prespecified clinical threshold of 0.1 logMAR. (Table 3).

**Table 3:** UDVA and CDVA (logMAR) Postoperative uncorrected (UDVA) and corrected (CDVA) distance visual acuity for enhanced monofocal and EDOF IOL groups are shown in logMAR values. Mean values, standard deviations and the number of eyes of each group are presented. Mean differences were calculated as enhanced monofocal minus EDOF, with corresponding 95% confidence intervals (CIs). A negative mean difference favors the enhanced monofocal and a positive mean difference favors the EDOF group. Lower logMAR values indicate better visual acuity [15-19]. UDVA: uncorrected distance visual acuity; CDVA: corrected distance visual acuity; EDOF: extended depth of focus

Studies	UDVA							CDVA						
	Enhanced monofocal			EDOF			Mean difference	Enhanced monofocal			EDOF			Mean difference
	Mean	SD	N	Mean	SD	N	(95% CI)	Mean	SD	N	Mean	SD	N	(95% CI)
Sabur [15]	0.05	0.08	36	0.06	0.08	40	-0.01 (-0.05, 0.03)	0.02	0.04	36	0.02	0.04	40	0.00 (-0.02, 0.02)
Lee [16]	0.04	0.06	48	0.07	0.11	40	-0.03 (-0.07, 0.01)	0.02	0.04	48	0.02	0.06	40	0.00 (-0.02, 0.02)
Corbelli [17]	0.01	0.02	50	0.02	0.05	50	-0.01 (-0.02, 0.00)	0.00	0.02	50	0.01	0.02	50	-0.01 (-0.02, 0.00)
Corbett [18]	N/A	N/A	114	N/A	N/A	120	N/A	0.05	0.08	114	0.06	0.08	120	-0.01 (-0.03, 0.01)
Jeon [19]	0.11	0.1	102	0.13	0.13	72	-0.02 (-0.06, 0.02)	0.03	0.05	102	0.05	0.08	72	-0.02 (-0.06, 0.02)

Intermediate visual acuity outcomes (UIVA and DCIVA) showed heterogeneity across the studies. Corbelli et al. reported a clinically meaningful advantage for EDOF IOL in both UIVA and DCIVA. The rest of the studies showed either no statistically significant difference or differences below the threshold for clinical relevance (Table 4).

**Table 4:** UIVA and DCIVA (logMAR) Postoperative uncorrected (UIVA) and corrected (DCIVA) intermediate visual acuity for enhanced monofocal and EDOF IOL groups are shown in logMAR values. Mean values, standard deviations and the number of eyes of each group are presented. Mean differences were calculated as enhanced monofocal minus EDOF, with corresponding 95% confidence intervals (CIs). A negative mean difference favors the enhanced monofocal and a positive mean difference favors the EDOF group. Lower logMAR values indicate better visual acuity. "N/A" indicates that the study did not assess this particular outcome [15-19]. UIVA: uncorrected intermediate visual acuity; DCIVA: distance corrected intermediate visual acuity; IOL: intraocular lens; EDOF: extended depth of focus

Studies	UIVA							DCIVA						
	Enhanced monofocal			EDOF			Mean difference	Enhanced monofocal			EDOF			Mean difference
	Mean	SD	N	Mean	SD	N	(95% CI)	Mean	SD	N	Mean	SD	N	(95% CI)
Sabur [15]	0.17	0.09	36	0.19	0.10	40	-0.02 (-0.06, 0.02)	0.17	0.08	36	0.18	0.09	40	-0.01 (-0.05, 0.03)
Lee [16]	0.16	0.12	48	0.19	0.17	40	-0.03 (-0.09, 0.03)	N/A	N/A	48	N/A	N/A	40	N/A
Corbelli [17]	0.28	0.06	50	0.03	0.05	50	0.25 (0.23, 0.27)	0.25	0.07	50	0.02	0.04	50	0.23 (0.21, 0.25)
Corbett [18]	N/A	N/A	114	N/A	N/A	120	N/A	0.18	0.14	114	0.13	0.08	120	0.05 (0.02, 0.08)
Jeon [19]	0.26	0.09	102	0.22	0.12	72	0.04 (0.01, 0.07)	N/A	N/A	102	N/A	N/A	72	N/A

For near visual acuity outcomes (UNVA and DCNVA), EDOF IOLs achieved statistically better performance compared to enhanced monofocal IOLs across all studies. However, differences of clinical significance were observed in only a subset of studies, with variable magnitude across outcomes (Table 5).

**Table 5:** UNVA and DCNVA (logMAR) Postoperative uncorrected (UNVA) and corrected (DCNVA) near visual acuity for enhanced monofocal and EDOF IOL groups are shown in logMAR values. Mean values, standard deviations and the number of eyes of each group are presented. Mean differences were calculated as enhanced monofocal minus EDOF, with corresponding 95% confidence intervals (CIs). A negative mean difference favors the enhanced monofocal, and a positive mean difference favors the EDOF group. Lower logMAR values indicate better visual acuity. "N/A" indicates that the study did not assess this particular outcome [15-19]. UNVA: uncorrected near visual acuity; DCNVA: distance corrected near visual acuity; IOL: intraocular lens; EDOF: extended depth of focus

Studies	UNVA							DCNVA						
	Enhanced monofocal			EDOF			Mean difference	Enhanced monofocal			EDOF			Mean difference
	Mean	SD	N	Mean	SD	N	(95% CI)	Mean	SD	N	Mean	SD	N	(95% CI)
Sabur [15]	0.50	0.09	36	0.31	0.12	40	0.19 (0.14, 0.24)	0.50	0.08	36	0.30	0.11	40	0.20 (0.16, 0.24)
Lee [16]	0.51	0.27	48	0.32	0.19	40	0.19 (0.09, 0.29)	N/A	N/A	48	N/A	N/A	40	N/A
Corbelli [17]	0.32	0.04	50	0.24	0.08	50	0.08 (0.06, 0.10)	0.32	0.04	50	0.26	0.08	50	0.06 (0.04, 0.08)
Corbett [18]	N/A	N/A	114	N/A	N/A	120	N/A	0.43	0.16	114	0.37	0.11	120	0.06 (0.03, 0.09)
Jeon [19]	0.42	0.11	102	0.32	0.11	72	0.10 (0.07, 0.13)	N/A	N/A	102	N/A	N/A	72	N/A

Due to heterogeneity in assessment tools, pooling was not feasible for glare and halo perception. Overall, these subjective photic phenomena were comparable between enhanced monofocal and EDOF IOLs in most studies. Corbelli et al. reported significantly fewer glare and halo symptoms with enhanced monofocal IOLs, while the remaining studies showed no significant between-group differences (Tables 6-9).

**Table 6:** Glare and halo perception in the Sabur study The table consists of two parts, the left one presenting the number of glares and the right one presenting the number of halos between each IOL group in the Sabur study [15]. "Events" indication is used to describe the number of eyes that experienced glares or halos, out of the "total" number of eyes of each group. Group comparisons were performed using the chi^2^ test. IOL: intraocular lens; EDOF: extended depth of focus

Study	Glares (events/total)	P: 0.850	Halos (events/total)	P: 0.819
	Enhanced monofocal	EDOF	Enhanced monofocal	EDOF
Sabur [15]	5/36	6/40	7/36	8/40

**Table 7:** Glare and halo perception: questionnaire scores in the Lee study Mean scores of a five-point scale questionnaire for glare and halo perception in the enhanced monofocal and EDOF groups are reported with standard deviations (SDs). Higher scores indicate greater severity of symptoms. Group comparisons were performed as reported in the original study [16]. EDOF: extended depth of focus

Study	Enhanced monofocal	EDOF	Enhanced monofocal	EDOF
	Glares (mean score ± SD)	P: 0.802	Halos (mean score ± SD)	P: 0.945
Lee [16]	2.2 ± 1.2	2.4 ± 1.4	2.0 ± 1.3	2.2 ± 1.5

**Table 8:** Glare and halo perception: NEI-RQL-42 questionnaire scores in the Corbelli study Mean NEI-RQL-42 questionnaire scores for glare and halo perception in the enhanced monofocal and EDOF groups are presented with standard deviations (SDs). Higher scores indicate better subjective quality of vision. Group comparisons were performed as reported in the original study [17]. NEI-RQL-42: National Eye Institute Refractive Error Quality of Life-42; EDOF: extended depth of focus

Study	Glares (mean NEI-RQL-42 score ± SD)	P: 0.0288	Halos (mean NEI-RQL-42 score ± SD)	P: 0.0079
	Enhanced monofocal	EDOF	Enhanced monofocal	EDOF
Corbelli [17]	67.0 ± 22.5	49.0 ± 26.5	74.0 ± 23.4	57.0 ± 19.8

**Table 9:** Glare and halo perception: Corbett study Frequency of no or rare glare and halo symptoms is reported using the Patient-Reported Visual Symptoms Questionnaire (PRVSQ) in the enhanced monofocal and EDOF groups. Results are presented as the number of patients reporting no or rare symptoms per group [18]. EDOF: extended depth of focus

Study	No or rare glares		No or rare halos	
	Enhanced monofocal	EDOF	Enhanced monofocal	EDOF
Corbett [18]	56/57	57/60	55/57	55/60

Results of Syntheses

Meta-analysis was attempted for all continuous outcomes (uncorrected and corrected distance, intermediate, and near visual acuities). However, it was only feasible for distance visual acuity (UDVA and CDVA), due to the detection of high heterogeneity in the other outcomes. Pooled analyses demonstrated statistically significant differences favoring enhanced monofocal IOLs for both UDVA and CDVA. However, effect sizes were below the prespecified threshold for clinical relevance. Heterogeneity was negligible in both analyses.

Subgroup analyses by EDOF optical design had been prespecified; however, they were not conducted because only one Acrysof IQ Vivity study and one Tecnis PureSee study were available, preventing meaningful comparison across subgroups (Tecnis Symfony (diffractive): three studies; Acrysof IQ Vivity (wavefront-shaping): one; Tecnis PureSee (refractive): one). Leave-one-out sensitivity analyses were performed for both UDVA and CDVA, resulting in no alteration of the clinical interpretation of the pooled results.

**Figure 3:**
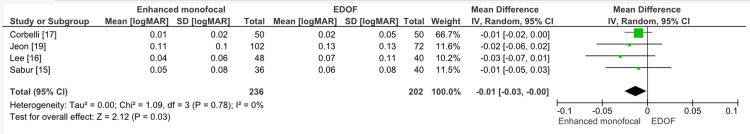
Forest plot comparing enhanced monofocal and extended depth of focus (EDOF) intraocular lenses for postoperative uncorrected distance visual acuity (UDVA) The random effects model was used for meta-analysis. The four studies [15-19] that assessed UDVA are included. LogMAR values are used. Means, standard deviations (SDs) and the number of eyes of each group are presented. Mean differences were calculated as enhanced monofocal minus EDOF, with corresponding 95% confidence intervals (CIs). Negative mean differences favor enhanced monofocal IOLs. Lower logMAR values indicate better visual acuity [15-19].

**Figure 4:**
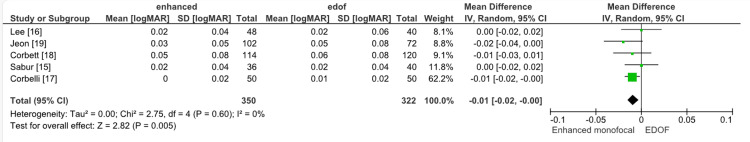
Forest plot comparing enhanced monofocal and extended depth of focus (EDOF) intraocular lenses for postoperative corrected distance visual acuity (CDVA) The random effects model was used for meta-analysis. All five studies, assessing CDVA, are included [15-19]. LogMAR values are used. Means, standard deviations (SDs) and the number of eyes of each group are presented. Mean differences were calculated as enhanced monofocal minus EDOF, with corresponding 95% confidence intervals (CIs). Negative mean differences favor enhanced monofocal IOLs. Lower logMAR values indicate better visual acuity [15-19].

Figures 3-4 present the forest plots for the main UDVA and CDVA meta-analyses, respectively.

Discussion

The aim of this systematic review and meta-analysis was to directly compare enhanced monofocal to EDOF IOLs by accumulating all available evidence from head-to-head comparative studies. It was evident that both IOL types achieved excellent UDVA and DCVA. Quantitative synthesis was feasible for these outcomes, and it demonstrated statistically significant differences favoring the enhanced monofocal IOLs; however, the magnitude of these differences was minimal and below the prespecified threshold for clinical relevance. This was further confirmed with leave-one-out sensitivity analyses.

Intermediate visual acuity outcomes varied across studies. Most studies reported no statistically significant difference between the two IOLs, while only Corbelli et al.'s study presented a better performance of the EDOF IOL, with a clinically meaningful difference in postoperative UIVA and DCIVA compared to the enhanced monofocal IOL. Differences in IOL design and study characteristics may have contributed to this variability in intermediate visual acuity outcomes. These heterogeneous findings suggest that intermediate visual performance may be driven more by the specific optical design of individual EDOF lenses than by the broad classification of EDOF versus enhanced monofocal IOLs.

Regarding UNVA and DCNVA, EDOF IOLs were consistently favored across all included studies. Nevertheless, clinically meaningful differences were noted only in three out of five studies, suggesting that the extent of near vision improvement provided with EDOF IOLs may vary among patients and lens designs.

Glare and halo perception was assessed in four out of five studies; the Corbelli study concluded that the enhanced monofocal IOL resulted in significantly less phenomena, while the rest of the studies showed an equally good postoperative quality of vision between the two groups. Heterogeneity in assessment tools did not allow quantitative pooling of these outcomes.

Supportive, yet not confirming, context for these results is provided by other studies. A scoping review conducted by Fernandez et al. in 2023 attempted to position enhanced monofocal IOLs in the IOL spectrum, compared to conventional monofocal and EDOF IOLs. It recorded no statistically significant difference in intermediate visual acuity between enhanced monofocal and EDOF IOLs, but a statistically significant difference in near visual acuity [21]. Scheepers and Hall performed a randomized controlled trial in 2023, comparing clinical visual outcomes achieved with two types of EDOF IOLs, Symfony and Acrysof IQ Vivity, with the latter resulting in significantly fewer glares and halos [22]. Moreover, a network meta-analysis comparing all types of IOL pertaining to presbyopia-correcting cataract surgery (monofocal, enhanced monofocal, trifocal, EDOF) was conducted by Li et al. in 2024. Indirect comparisons suggested the superiority of EDOF in intermediate visual acuity, while enhanced monofocal IOLs preserved excellent distance visual quality and had a low rate of photic phenomena [23]. Finally, a retrospective real-world comparative study by Kim et al., comparing TECNIS PureSee (EDOF) to Tecnis Eyhance, was published in July 2025, after our search was executed. The EDOF IOL demonstrated better uncorrected and near visual acuity, with comparable distance visual acuity and contrast sensitivity between the two groups [24].

The evidence that was gathered in our systematic review was sufficient for quantitative synthesis pertaining to distance visual acuity. However, meta-analysis was not feasible for intermediate and near visual acuities due to high heterogeneity (I^2^ > 50%); therefore, the results were attempted to be synthesized. In all of the studies included in our review, patient characteristics and tools of outcome measurement were similar. However, there were several circumstances that may have contributed to heterogeneity. First, different EDOF optical designs were used across the studies (diffractive EDOF, wavefront-shaping, refractive), which may influence intermediate visual acuity. There was only one unilateral study; four studies were bilateral, which highlights the probability of within-patient clustering. These factors, which have a stronger effect on intermediate vision than distance vision, created heterogeneity too great to permit a valid meta-analysis.

This study has several limitations. To begin with, the number of available studies was relatively small, and most of them were non-randomized. The small number of included studies, in particular, along with the modest sample sizes among some of them, could increase susceptibility to small-study effects. Moreover, follow-up in all studies included in our review was adequate for assessing post-operative best corrected visual acuity; however, three to six-month time frames could possibly provide less reliable results than longer-term follow-ups. Our search strategy attempted to include all up-to-date evidence; nevertheless, given also the language restriction, omissions may have been made. In addition, within-patient clustering was not accounted for; treating both eyes from the same patient as independent observations may have led to underestimation of variance and overstatement of statistical significance. Furthermore, subgroup analysis by EDOF type was not applicable due to the small number of studies. Finally, there is a risk of publication bias, given the restricted body of literature and the existence of several industry-sponsored studies.

## Conclusions

Overall, our findings suggest that both enhanced monofocal and EDOF IOLs achieve excellent distance visual acuity, with minimal and clinically insignificant differences between them. EDOF IOLs tend to provide better intermediate and near visual acuity; however, this statistically observed trend is heterogeneous and translates into clinically meaningful benefit in only a subset of studies. Quantitative synthesis was not feasible for intermediate and near visual acuity outcomes due to substantial heterogeneity across studies. Generally, both IOL types led to favorable results in terms of glare and halo perception.

Given the limited number of direct comparative studies, along with the predominance of non-randomized study designs in our review and the relatively short-term follow-ups, these findings should be interpreted cautiously. Rather than supporting a universal recommendation for one lens category over another, the results highlight the importance of individualized IOL selection. Further head-to-head comparative studies incorporating standardized intermediate vision metrics, defocus curves, contrast sensitivity, and validated patient-reported outcome measures are needed to better position enhanced monofocal and EDOF IOLs in clinical practice.

## Appendices

**Table 10:** Expanded JBI risk of bias assessment for non-randomized studies Domain-level assessments using the Joanna Briggs Institute (JBI) checklists for non-randomized studies are presented. Each domain is rated as “Yes” (Y), “No” (N), “Unclear” (U) or not applicable (N/A), with the respective justifications and numerical totals provided descriptively. The “Included in meta-analysis” column reflects the prespecified threshold of 50% of domains rated “Yes”, applied consistently for quantitative synthesis [15-17,19].

Study	Domain question	Answer	Justification	Total score	Included in meta-analysis
Sabur [15]	1) Were the groups comparable other than presence/absence of disease?	U	Apart from inclusion/exclusion criteria for surgery and ocular history, no further demographic details were reported.	5/10	Yes
	2) Were cases and controls matched appropriately?	U	Groups similar in age, gender, preoperative data; no further detail on source population		
	3) Same criteria used for identification of cases/controls?	Y	All underwent bilateral phacoemulsification by same surgeon; exclusion criteria clear.		
	4) Was exposure measured in a standard, valid and reliable way?	Y	Preoperative exam, surgery, postoperative follow-up adequately described.		
	5) Was exposure measured the same way for cases and controls?	Y	Procedure was identical for both groups.		
	6) Were confounding factors identified?	U	Ocular history reported; comorbidities not described.		
	7) Were strategies to deal with confounding stated?	N	No strategy described.		
	8) Were outcomes assessed in a standard, valid and reliable way?	Y	Uncorrected intermediate visual acuity measured with Jaeger charts and converted to logMAR.		
	9) Was exposure period long enough to be meaningful?	Y	Three-month follow-up sufficient for assessment.		
	10) Was appropriate statistical analysis used?	U	Descriptive stats OK; confounder analysis missing.		
Lee [16]	1) Clear inclusion criteria?	Y	Adequate criteria for age, ocular history, preop astigmatism, potential postop CDVA.	8/10	Yes
	2) Condition measured reliably for all participants?	Y	Surgical technique and exams standard for all.		
	3) Valid methods for identifying condition?	Y	LogMAR chart used.		
	4) Consecutive inclusion of participants?	Y	All patients within a defined time frame included.		
	5) Complete inclusion of participants?	Y	All completed 3-month follow-up.		
	6) Clear demographics reported?	Y	Age, gender, ethnicity reported.		
	7) Clear clinical info reported?	U	Preoperative exams described; cataract stage/systemic history not detailed.		
	8) Outcomes/follow-up clearly reported?	Y	Uncorrected intermediate visual acuity reported in charts/figures.		
	9) Presenting site demographics clear?	U	Ethnicity and gender ratio reported, but correlation with outcomes unclear.		
	10) Statistical analysis appropriate?	Y	Software and tests suitable.		
Corbelli [17]	1) Clear inclusion criteria?	U	Exclusion criteria clear; inclusion criteria insufficiently detailed.	6/10	Yes
	2) Condition measured reliably?	Y	Standard surgical technique and exams.		
	3) Valid methods for identification?	Y	LogMAR chart used.		
	4) Consecutive inclusion?	Y	All patients included within defined timeframe.		
	5) Complete inclusion?	Y	All completed 6-month follow-up.		
	6) Clear demographics?	U	Only age clearly reported.		
	7) Clear clinical info?	U	Preoperative exams reported; cataract stage/systemic history missing.		
	8) Outcomes/follow-up clearly reported?	Y	Postoperative UIVA reported in charts/figures.		
	9) Presenting site demographics clear?	U	Only age reported.		
	10) Statistical analysis appropriate?	Y	Software and tests suitable.		
Jeon [19]	1) Two groups similar, same population?	Y	All data from same hospital; inclusion/exclusion criteria clear.	6/11	Yes
	2) Exposure measured similarly for both groups?	Y	Same surgeon and procedure for all.		
	3) Exposure measured validly/reliably?	Y	Preop exam, surgery, follow-up described.		
	4) Confounding factors identified?	U	Ocular history reported; comorbidities unclear.		
	5) Strategies to deal with confounding?	U	T-tests/chi-square used; no further confounder control.		
	6) Groups free of outcome at start?	N	Retrospective; patients had already undergone cataract surgery.		
	7) Outcomes measured validly/reliably?	Y	VA measured with ETDRS, converted to logMAR.		
	8) Follow-up time sufficient?	Y	3 months adequate.		
	9) Follow-up complete?	Y	Data only for patients completing follow-up.		
	10) Strategies for incomplete follow-up?	N/A	Not applicable.		
	11) Statistical analysis appropriate?	U	Descriptive statistics OK; confounders not fully addressed.		
